# The Organism of Lung-plague

**Published:** 1899-01

**Authors:** 


### THE ORGANISM OF LUNG-PLAGUE.

*A review and criticism by Professor J. W. Schütz of a paper presented by Nocard and Roux at the meeting of the Central Society of Veterinary Medicine of Paris on March* 24, 1898.

TRANSLATED BY LEONARD PEARSON, B.S., V.M.D.,
DEAN, VETERINARY DEPARTMENT, UNIVERSITY OF PENNSYLVANIA, PHILADELPHIA,

From the Archiv für Wissenschaftliche und Praktische Thierheilkunde, 1898, Heft v.

THE albuminous, yellow, and clear fluid which fills the interstitial tissue of the lungs in a case of lung-plague (contagious pleuropneumonia of cattle) is very virulent. When a drop of this fluid is injected under the skin of a healthy ox there results, in from eight to twenty-five days, a painful, hot swelling of variable size on the spot at which the injection was made. If the injection is made under the skin of the trunk the animal becomes feverish, and the swelling at the point of injection spreads rapidly, and sometimes causes the death of the animal. By post-mortem examination it is found that the connective-tissue spaces contain considerable yellow, clear fluid, which in some areas is consolidated into gelatinous masses. The inflammatory process never reaches the lungs. One finds at most a small quantity of fluid exudate in the pleural sacs. The death of the animal must, therefore, result from poisoning. Many animals thus inoculated recover. In these, after a few days, the further enlargement of the swelling ceases, and it afterward gradually disappears. Such animals are immune. This favorable result of the injection occurs regularly when it is made at a point as far from the trunk as possible; for example, on the end of the tail, where there is strong subcutaneous tissue and where the low temperature is not so well adapted to a rapid growth of the infectious principle of lung-plague; but even a small swelling of the tail is sufficient to make the animal immune. Sometimes the tension of the tissue, as a result of too profuse development, is so great that part of the tail is destroyed. Occasionally the swelling extends beyond the point of injection in the direction of the tail, and when it reaches the trunk death usually results.

Goats, sheep, dogs, pigs, rabbits, guinea-pigs, and fowls can receive injections of this fluid exudate from the connective tissue of the diseased lungs into the subcutaneous tissue or the abdominal cavity without injury. This fact was known to Willems in 1850, and his method of preventive inoculation is based upon it. But an objection to this method is that it calls for lymph obtained from diseased lungs, which changes easily, ferments, and thereby loses its virulence. Therefore, the lung of an animal afflicted with lung-plague must be obtained when it is quite fresh, if lymph is to be collected for inoculation. Sometimes it happens that in such a lung only the old lesions exist, and then the fluid exudate has lost its virulence or has disappeared, or it may be that there are no suitable cases at hand that can be killed for this purpose.

A great step in the production of this lymph was made when Pasteur inoculated calves subcutaneously with the fluid exudate from a diseased lung. Thereby a source from which lymph could be obtained was discovered. But even this is not sufficient, because the fluid exudate from the lungs, even when it is perfectly pure, loses its virulence in from four to six weeks, and is then no longer suitable for inoculation; so in order to have good lymph ready at all times it was necessary to establish places for its production where new animals could be inoculated every four weeks. This has been done in some countries.

All these difficulties would have been overcome if it had been possible to isolate and cultivate the cause of lung-plague. Until recently the authors have not been able to do this, although they used every known culture-material and method for this purpose. At last they succeeded in making such cultures *in vivo* by the help of the collodion sacks that were first used by Metchnikoff, Roux, and Salimbeni in their investigations of the toxins and antitoxins of cholera. These cultures were made as follows: A small sack is made of collodion; this is sterilized and filled with a few cubic centimetres of bouillon, which is seeded with the virulent fluid that is to be examined. Then these sacks are sealed and placed in the abdominal cavity of an animal. At the expiration of from a few days to several months, according to the nature of the microörganism that is under investigation, the animal is killed. By the post-mortem examination it is possible to find the sacks in some part of the abdominal cavity, where they are enclosed by fibrin and cells. The wall of the little sack prevents the passage of the microorganisms into the abdominal cavity, and in the same way the passage of the cells from the abdominal cavity into the bouillon is prevented. On the other hand, the wall of the sack permits the exchange of dissolved substances, so that the chemical composition of the fluid in the sack can change, and toxins that are produced by the microörganisms can pass out and cause symptoms of intoxication or even the death of the animal. If the materials that pass into the bouillon are suitable for the growth of the microörganism, cultures of this will develop.

It was by this method that the authors attempted to cultivate the microörganisms of lung-plague. Collodion sacks were filled with bouillon that had been seeded with fluid from the diseased lungs; then the sacks were sealed and placed in the abdominal cavities of rabbits. In from fifteen to twenty days the contents of the sacks became clouded, but they contained neither cells nor bacteria that grow in ordinary bouillon. But the fluid contained inconceivable numbers of light-refracting, motile bodies that are so small that their form could not be ascertained even after they were stained. It was found that when a collodion sack filled with nothing but bouillon was placed in the abdominal cavity of the same rabbit the fluid was not changed, and remained clear. According to the view of the authors, these small, light-refracting bodies that grow in the seeded bouillon are living organisms. These growths occur in the abdominal cavity under the favorable conditions that have been described—that is, when the bodies in the sacks are protected from the destructive action of the phagocytes.

The following experiments tend to confirm this view: If one places a collodion sack that is filled with bouillon seeded with the cloudy fluid mentioned above in the peritoneal cavity of a rabbit, and also places another sack similarly filled with seeded bouillon in the peritoneal cavity, the latter sack having, however, been heated before its insertion, it is found that the contents of the heated sack will remain clear and transparent, while the other becomes cloudy, as a result of the growth of the above-mentioned bodies—that is, the organisms are killed by heat. The cloudy contents of the sack containing the growing culture can be used to seed other bouillon, and so on.

The rabbits that carried the sacks in their abdominal cavities were thin at the time they were killed. Some even died of cachexia. The autopsy showed no alterations, and the blood and organs were sterile, so it is evident that a poisoning was caused by the products of the organisms growing in the sacks. On the other hand, rabbits remained healthy when sacs containing nothing but bouillon were placed in their peritoneal cavities. It also happened that rabbits emaciated more rapidly when large sacks or several of them were placed in their peritoneal cavities. While rabbits are immune to the bacteria of lung-plague, they succumb to the action of its toxins. These experiments did not succeed on guinea-pigs.

Five cows were inoculated with a small quantity of the fluid contents of the sacks, and after this the characteristic lung-plague inoculation swelling developed. One cow died as the result of the extensive swelling. The other four cows recovered fully. Two of these became immune, as was shown by subsequent inoculation with one c.c. of the fluid exudate from a diseased lung. That the material used for this inoculation was virulent was shown by the fact that another cow inoculated with ten drops of the same exudate died. The third cow was found to be immune when she was inoculated three months later. The fourth cow has not yet been tested in this way.

If one tries to make aërobic or anaërobic cultures on other media from the cultures contained in the collodion sacks that have been in the rabbits fifteen or twenty days, it is found that no growth occurs.

These attempts succeed if one uses the unseeded and sterilized bouillon that has been in collodion sacks in the peritoneal cavities of cows or rabbits for several weeks. Under these conditions the chemical composition of the bouillon is changed; it becomes richer in albumin, and is then adapted to the growth of the organism of lung-plague. If such bouillon is seeded it becomes cloudy after a sojourn of seventy-two hours in the incubator, and contains large numbers of small, refractive, motile bodies.

At length the authors found a culture-medium upon which these organisms could be grown without difficulty. This substance consists of a solution of peptone to which a small quantity of rabbit-serum has been added. The peptone solution is to be prepared according to the method of Martin, and to five c.c. of this solution four drops of serum are added. The organism of lung-plague grows in this solution in the presence of air, and it is quite the same whether this nutritive medium is seeded with the cultures grown in the collodion sacks or with the fluid exudate of the diseased lungs of cattle.

The growth of the organism in this nutritive fluid has the advantage that the virulence is retained, while, on the other hand, it appears to be gradually lost by cultivating it in the abdominal cavity of a rabbit. However, the pure cultures shonld be studied and their virulence tested. It is to be noted that animals of the same age and breed possess different degrees of susceptibilty for the infectious material of lung-plague, and, therefore, the question as to whether this material has undergone reduction in virulence or not can only be settled by a large series of experiments. That the infectious material in the culture remains virulent is shown by the fact that a cow inoculated on the 26th of February, 1898, with ten drops from a culture of the sixth generation of the organism of lung-plague died on the 19th of March from the great swelling following the injection.

The authors believe that the method here described can be used for discovering other, as yet unknown, organisms of some other diseases. The discovery of the organism of lung-plague was made difficult by two conditions: first, its very small size; and, second, the difficulty of propagating it in cultures. This work makes it probable that there are still smaller microörganisms that cannot be determined by present methods, but it may be possible to cultivate even these organisms, and perhaps without any visible change in the nutritive fluid. In spite of the fact that the organism of lung-plague grows luxuriantly, the cloudiness of the nutritive fluid is very slight, and sometimes can only be determined by comparing the culture with unseeded bouillon. Multiplication is the only sign by which such organisms can be recognized, therefore it may be that cultures of such microörganisms have been made but not observed.

### *Conclusions.*

The organism of lung-plague is exceedingly small, smaller than any of the microörganisms previously known, and so small that its form cannot be determined even after it is stained.

This organism grows in collodion sacks that are placed in the abdominal cavity of a rabbit.

It does not grow under ordinary conditions.

It can only be grown in peptone-bouillon to which the serum of cows or rabbits has been added in the proportion of twenty-five to one.

